# Route-dependent dissemination with conserved blood–tumor barrier ultrastructure in intracranial metastasis models

**DOI:** 10.1038/s41598-026-37760-z

**Published:** 2026-03-14

**Authors:** Jian Zhao, Yuehua Zhang, Zhigong Wei, Kai Li, Dan Li, Yongsheng Wang

**Affiliations:** 1 Thoracic Oncology Ward, Cancer Center, and State Key Laboratory of Biotherapy, West China Hospital, Sichuan University, Chengdu, 610041 Sichuan People’s Republic of China; 2 West China School of Public Health and West China Fourth Hospital, Sichuan University, Chengdu, 610041 Sichuan People’s Republic of China; 3 Department of Biotherapy, Cancer Center and State Key Laboratory of Biotherapy, West China Hospital, Sichuan University, Chengdu, 610041 Sichuan People’s Republic of China; 4 Institute of Respiratory Health, Frontiers Science Center for Disease-Related Molecular Network, and Precision Medicine Research Center, Precision Medicine Key Laboratory of Sichuan Province, West China Hospital, Sichuan University, Chengdu, 610041 Sichuan People’s Republic of China

**Keywords:** Brain metastasis, Neurovascular unit, Blood-tumor barrier, Animal model, Ultrastructure, Electron microscopy, Cancer, Neuroscience, Oncology

## Abstract

The dissemination route of brain metastases dictates their spatiotemporal distribution, but whether it alters the ultrastructural state of the blood-tumor barrier remains unresolved. Here, we compared a modified hematogenous dissemination model that minimizes extracranial signal confounds with a direct intracranial inoculation model. The hematogenous model produced multifocal brain lesions, whereas the intracranial model formed unifocal masses. Longitudinal bioluminescence imaging revealed significantly different growth rates between models, yet no statistically significant difference in overall survival was detected under the current cohort sizes and humane endpoints. Critically, exploratory transmission electron microscopy at the tumor-brain interface revealed a conserved pathological phenotype of the blood-tumor barrier in established lesions, irrespective of the seeding route. This pattern was characterized by endothelial cell swelling, a discontinuous basement membrane, and retraction of astrocytic end-feet. These findings suggest that while the seeding route determines the macroscopic pattern of disease, the established brain microenvironment imposes a stereotyped mode of neurovascular unit failure. This work provides a methodologically refined platform for studying brain metastasis and presents direct ultrastructural evidence consistent with a conserved blood-tumor barrier pathology, outlining a testable conceptual framework. These hypothesis-generating ultrastructural observations, derived from representative specimens, warrant quantitative validation.

**Supplementary Information:**

The online version contains supplementary material available at 10.1038/s41598-026-37760-z.

## Introduction

The central nervous system (CNS) operates within a meticulously controlled microenvironment, a state of homeostasis essential for high-fidelity synaptic transmission^1^. The primary guardian of this environment is the blood–brain barrier (BBB), a dynamic interface segregating the neural parenchyma from the systemic circulation^2^. The modern understanding of this barrier is embodied in the concept of the neurovascular unit (NVU)—a complex, multicellular network of brain microvascular endothelial cells, pericytes, astrocytes, microglia, and neurons^3^. The NVU is a co-evolved, self-assembling system where the function of each component is contingent upon signals from the others^4^. This interdependence explains why an insult to any single element can trigger a cascading dysfunction across the entire unit.

In the context of brain metastasis (BrM), the NVU and its perivascular niche represent the primary battlefield where the fate of tumor cells is decided^5^. Disseminated tumor cells (“seed”) are critically dependent on the neurovascular niche as the essential "soil," with a predominant initial growth strategy being vascular co-option, whereby cells hijack the pre-existing vasculature^6^. The vascular basement membrane (VBM) of the NVU provides an indispensable substrate for the adhesion, survival, and proliferation of carcinoma cells^7^. This interaction is not merely passive; metastatic cells appear to exploit the pre-existing biological logic of the neurovascular niche, behaving as pathological mimics of endogenous stem cells to colonize this foreign environment^8^.

To decipher this process, researchers rely on preclinical models where the choice of inoculation route fundamentally shapes the initial tumor-NVU interaction^9,10^. Direct intracranial (IC) injection creates a reproducible, unifocal “trauma-plus-tumor” insult, but introduces iatrogenic BBB disruption and acute inflammation^11^. In contrast, intracarotid artery (ICA) injection models a hematogenous “embolic-plus-tumor” insult^12^. However, conventional ICA models are notoriously confounded by extracranial tumor growth that contaminates imaging signals and complicates representative sampling^13^. A detailed comparison of the features, advantages, and limitations of these principal models is provided in Supplementary Table S1.

This methodological divergence raises a central scientific question: given these different initial insults, does the pathological response of the NVU ultimately diverge, or does it converge toward a common ultrastructural phenotype imposed by the brain microenvironment? To address this, we first developed a modified ICA (m-ICA) technique that reduces extracranial contributions to imaging and sampling, enabling brain-restricted longitudinal readouts. We then used this platform in a direct comparison with the IC model to test the hypothesis that once metastases are established, the potent remodeling signals of the local microenvironment will drive the NVU toward a conserved pathological ultrastructure. Here we focus on established lesions and provide qualitative ultrastructural comparisons to contextualize growth dynamics.

## Results

### The conventional ICA model is confounded by significant extracranial tumor growth

**Fig. 1:**
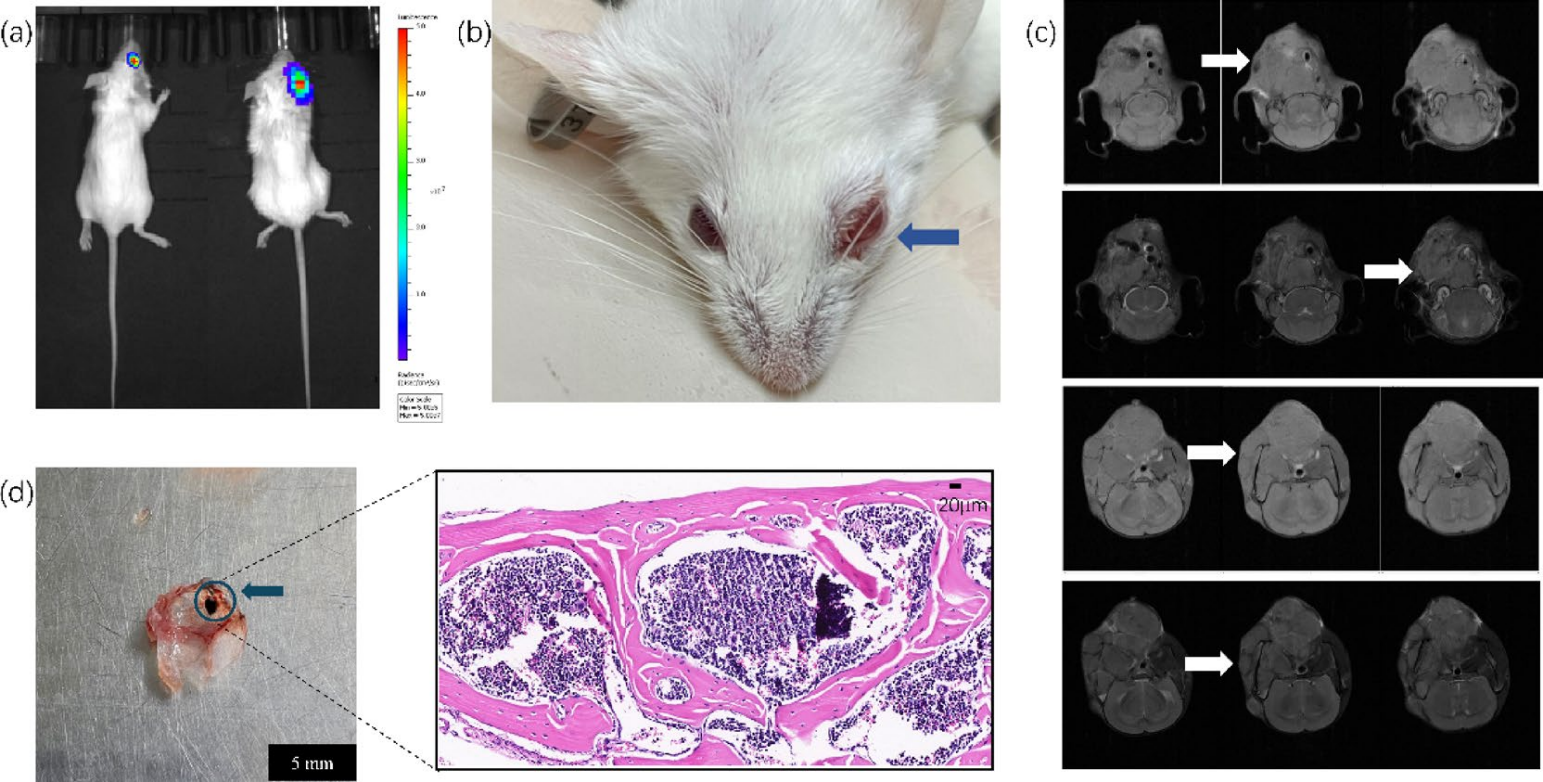
The conventional intracarotid artery (ICA) injection model is confounded by significant extracranial tumor growth. (**a**) Representative in vivo bioluminescence imaging (BLI) of two mice following conventional ICA injection, demonstrating strong signals localized predominantly to the orbital regions. (**b**) Gross photograph of a representative mouse exhibiting severe contralateral (left) ocular metastasis (blue arrow). The occurrence of contralateral lesions despite right-sided injection illustrates the extensive hemodynamic shunting across the Circle of Willis inherent to the conventional model. (**c**) Serial T2-weighted magnetic resonance images (MRI) of a mouse head. The white arrows indicate a large extracranial tumor mass in the ipsilateral (right) maxillofacial soft tissues. Note that while Fig. 1b shows a contralateral lesion, Fig. 1c shows an ipsilateral one, highlighting the variable dissemination inherent to the conventional model. (**d**) Gross pathology of a pigmented melanoma lesion on the skull (left panel, blue arrow) and corresponding Hematoxylin and Eosin (H&E) staining (right panel) confirming extensive tumor cell infiltration. Scale bars = 5 mm (left), 20 µm (right).

We first validated the well-documented limitation of the conventional ICA injection model. Following this procedure, in vivo BLI revealed strong signals localized predominantly to the orbital and maxillofacial regions, obscuring any potential intracranial signal (Fig. 1a, b). T2-weighted MRI and gross pathology confirmed the presence of large extracranial tumor masses (Fig. 1c, d). These data indicate that, under our conditions, conventional ICA was confounded by extracranial growth and did not permit reliable brain-specific longitudinal quantification.

### Modified ICA and IC models provide complementary platforms for brain metastasis research

**Fig. 2:**
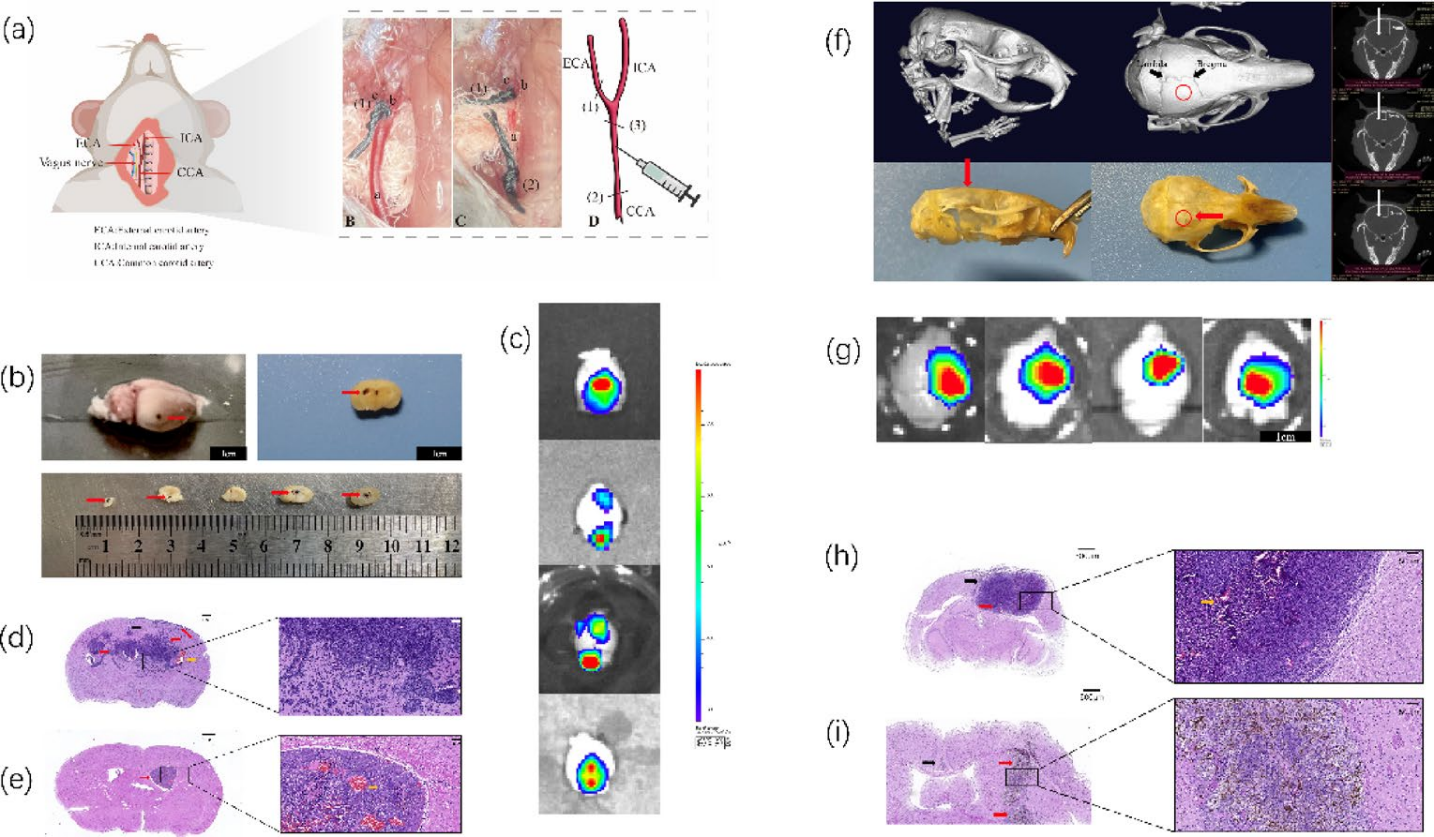
Establishment and comparative pathology of a modified hematogenous (m-ICA) and a direct intracranial (IC) brain metastasis model. (**a**) Schematic diagram (left) and intraoperative photographs (right) illustrating the modified intracarotid artery (m-ICA) injection technique, featuring the surgical exposure of the carotid bifurcation and the critical step of external carotid artery (ECA) ligation prior to injection into the common carotid artery (CCA). (**b**) Gross pathology of a representative brain with B16 melanoma metastases following m-ICA injection (top), and corresponding serial coronal sections revealing multiple, disseminated hemorrhagic lesions throughout the brain parenchyma (bottom). (**c**) Representative ex vivo bioluminescence imaging (BLI) of brains harvested after m-ICA injection, demonstrating the characteristic multifocal metastatic pattern, ranging from single foci to multiple and confluent lesions. (**d**, **e**) Representative H&E staining of coronal brain sections from the m-ICA model. (**d**) 4T1 breast cancer model showing multifocal tumor nodules in the parenchyma and meninges (red arrows). The yellow arrow indicates a focus of intratumoral hemorrhage, and the black arrow indicates a disseminated lesion extending to the midline. (**e**) B16 melanoma model showing infiltrative tumor nests (red arrows) and prominent intratumoral hemorrhage (yellow arrow). (**f**) Depiction of the stereotactic intracranial (IC) injection model. The red circle (dorsal view) and red arrow (lateral view) indicate the stereotactic injection target coordinates. Black arrows indicate the anatomical landmarks Bregma and Lambda. (**g**) Representative ex vivo BLI of brains harvested after IC injection, showing a single, large, unifocal tumor mass. (**h**, **i**) Representative H&E staining of coronal brain sections from the IC model. (**h**) A large 4T1 tumor mass (red arrow) displaying expansive growth. The yellow arrow indicates intratumoral hemorrhage, and the black arrow points to tumor extension caused by reflux along the needle track. (**i**) B16 melanoma showing a lobulated tumor mass (red arrow). Black arrows indicate focal seeding at the cortical surface caused by reflux along the needle track. Note that these needle-track associated features are inherent technical artifacts of the direct inoculation method. Scale bars in D, E, H, I = 500 µm (left panels), 50 µm (right panels).

To overcome this limitation, we implemented a modified ICA (m-ICA) technique, which involves the ligation of the external carotid artery prior to cell injection (Fig. 2a). This refinement effectively minimized confounding extracranial signals. Gross pathology, ex vivo BLI, and H&E staining confirmed that the m-ICA procedure resulted in a multifocal, disseminated pattern of metastases throughout the brain parenchyma in both B16 and 4T1 tumor systems (Fig. 2b–e). In parallel, the stereotactic IC model consistently produced a single, well-demarcated, unifocal tumor mass at the injection site (Fig. 2f–i). At the experimental endpoints, H&E analysis showed these unifocal IC tumors reached an approximate average diameter of 2.5 mm (4T1 model, Fig. 2h) and 2.0 mm (B16 model, Fig. 2i). In stark contrast, the m-ICA model produced disseminated, multifocal lesions of variable sizes (Fig. 2b, d, e). In the B16 model, these nodules had diameters up to 1.6 mm (Fig. 2e). In the 4T1 model, the lesions exhibited a highly infiltrative phenotype, frequently fusing to form larger, confluent tumor burdens with indistinct margins (Fig. 2d). In contrast, B16 melanoma lesions, while also multifocal, typically displayed a more expansive, lobulated growth pattern with relatively well-demarcated borders (Fig. 2e), consistent with the distinct co-optive growth mechanisms often observed in melanoma brain metastasis^14^. Conversely, the expansive growth phenotype of the IC tumors (Fig. 2h, i) is likely attributable to the mass effect generated by the stereotactic bolus injection^10^. Together, these optimized m-ICA and IC models provide two methodologically refined and anatomically distinct platforms for comparative analysis.

### Seeding strategy dictates macroscopic growth kinetics but not overall survival

**Fig. 3:**
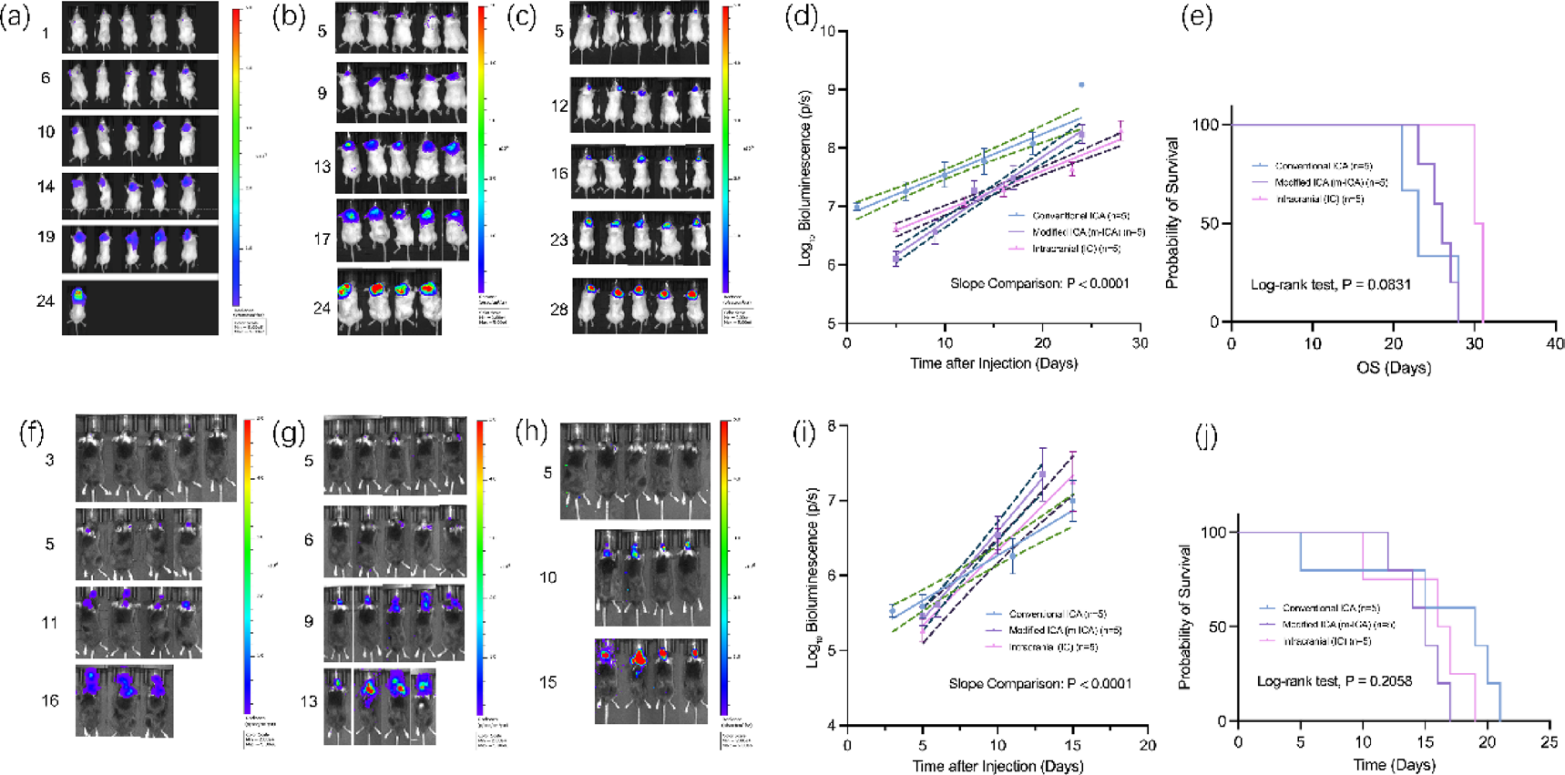
Seeding strategy dictates tumor progression dynamics but not overall survival. (**a**–**e**) Analysis of the 4T1/BALB/c breast cancer model. (**a**–**c**) Representative longitudinal in vivo bioluminescence imaging (BLI) of tumor growth in the conventional ICA (**a**), m-ICA (**b**), and IC (**c**) models over time. (**d**) Linear regression analysis of log-transformed BLI data. The slope of the regression line, representing the tumor growth rate, was significantly different among the three groups (F-test for slope comparison, *P* < 0.0001). (**e**) Kaplan–Meier survival analysis for the three models, showing no statistically significant difference in median overall survival (OS) (Log-rank test, *P* = 0.0831). (**f**-**j**) Corresponding analysis of the B16/C57BL/6 melanoma model. (**f**–**h**) Representative longitudinal BLI of tumor growth in the conventional ICA (**f**), m-ICA (**g**), and IC (**h**) models. (**i**) Linear regression analysis of log-transformed BLI data, demonstrating a significant difference in tumor growth rates among the groups (*P* < 0.0001). (**j**) Kaplan–Meier survival analysis, with no significant difference observed in median OS (Log-rank test, *P* = 0.2058). Data in (**d**) and (**i**) are presented as individual regression lines with mean ± 95% confidence intervals.

Longitudinal BLI monitoring revealed that the seeding strategy dictates macroscopic tumor progression. In both the 4T1/BALB/c (Fig. 3a–c) and B16/C57BL/6 (Fig. 3f–h) systems, the multifocal tumors established via m-ICA exhibited distinct growth trajectories compared to the unifocal tumors from IC inoculation. Linear regression analysis of log-transformed BLI data confirmed that the tumor growth rates were significantly different among the groups (F-test, *P* < 0.0001 for both systems) (Fig. 3d, i). Despite these significant differences in tumor growth dynamics, Kaplan–Meier analysis showed no statistically significant difference in median overall survival between the m-ICA and IC groups in either tumor system (log-rank *P* = 0.0831 and 0.2058, respectively) (Fig. 3e, j). Non-significant OS differences reflect failure to reject the null under current sample sizes and humane endpoints and should not be construed as evidence of equivalence.

### A Conserved BTB ultrastructural phenotype in established B16 brain metastases

**Fig. 4:**
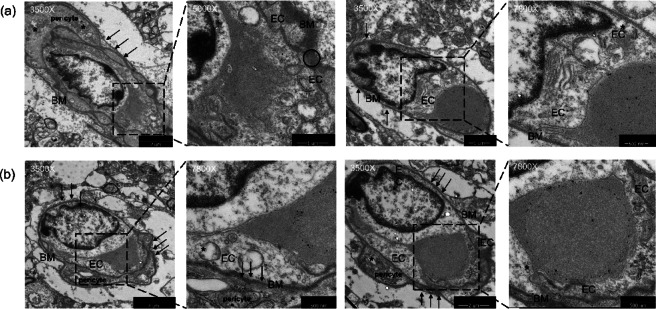
The ultrastructure of the blood-tumor barrier (BTB) is pathologically conserved across hematogenous and direct inoculation models. Transmission electron microscopy (TEM) was performed on the tumor-brain interface of established metastases. (**a**) Representative TEM images from the hematogenous m-ICA model. (**b**) Representative TEM images from the direct intracranial (IC) model. Despite the different seeding mechanisms, both models exhibit a remarkably conserved pathological phenotype. Key features include swollen endothelial cells (EC) containing cytoplasmic vacuoles (asterisks) indicative of aberrant vesicular transport activity. The basement membrane (BM) is attenuated, irregular, and appears discontinuous in multiple locations. Pericytes are visible within the compromised basement membrane. Critically, the perivascular astrocytic end-feet, which normally form a tight sheath around the vessel, are either completely lost or significantly retracted (indicated by arrows), creating an abnormal perivascular space. This demonstrates that once established in the brain parenchyma, metastases induce a convergent, structurally compromised BTB, regardless of their route of arrival. Labels: BM, basement membrane; EC, endothelial cell. Magnifications and scale bars are as indicated in each micrograph.

The central question of our study was whether the macroscopic differences would translate to distinct ultrastructural pathologies. To address this, we performed targeted TEM on established B16 melanoma metastases at the tumor-brain interface (Fig. 4). Remarkably, despite the disparate seeding mechanisms, we observed a strikingly conserved pathological phenotype of the blood-tumor barrier (BTB) in samples from both the m-ICA (Fig. 4a) and IC (Fig. 4b) models. Key recurring features included swollen endothelial cells (EC) containing cytoplasmic vacuoles (asterisks) indicative of aberrant vesicular transport activity, an attenuated and discontinuous basement membrane (BM), and profound retraction of perivascular astrocytic end-feet (arrows), creating an abnormal perivascular space. This qualitative observation provides direct ultrastructural evidence consistent with a conserved BTB pathology in established lesions across seeding routes in this model system.

## Discussion

This study addressed whether the ultrastructure of the neurovascular unit (NVU) in established brain metastases is dictated by the seeding route or converges on a pathological state imposed by the brain microenvironment. Methodologically, we implemented a modified intracarotid (m-ICA) model that reduces extracranial tumor burden^13,15^, thereby enabling brain-restricted attribution of longitudinal BLI signals. Despite macroscopic differences in lesion pattern and growth kinetics, our exploratory TEM analysis in the B16 melanoma model revealed a conserved BTB phenotype in established lesions. It is noteworthy that although the two models exhibit distinct macroscopic invasion borders driven by their seeding mechanics (infiltrative vs. expansive), our ultrastructural analysis reveals that the internal NVU remodeling converges to a shared pathological phenotype.

Regarding the macroscopic distribution mechanisms, although both models involved right-sided inoculations, they resulted in distinct patterns. The bilateral multifocal lesions in the m-ICA model result from hemodynamic shunting across the Circle of Willis during the injection bolus, supported by the recruitment of collateral pathways in the murine cerebral circulation^16^. In contrast, the IC model typically produces a unifocal mass^10,17^; however, extensive tumor growth can lead to significant midline shift or distinct surface nodules. We interpret these surface features as consequences of reflux along the needle track—a known technical risk of direct inoculation—rather than true hematogenous dissemination.

### A conserved pathophenotype: a stereotyped failure mode of the NVU

Our central finding is that distinct seeding routes culminate in a common pattern of NVU ultrastructural remodeling. These observations are consistent with the notion that the NVU may exhibit a limited repertoire of maladaptive responses to severe, sustained injury^18^. The pathological phenotype we observed is morphologically similar to features reported across multiple CNS conditions (e.g., stroke, TBI, MS, Alzheimer’s), and is consistent with findings of complex molecular alterations, such as widespread neurotransmitter receptor imbalances found in focal epilepsy^19,20^. For instance, enhanced endothelial transcytosis is an early leakage mechanism in stroke^21^, and astrocytic end-foot pathology is a hallmark of chronic multiple sclerosis lesions^22^. We note that morphological similarity does not imply shared mechanisms; targeted perturbation and multi-omic analyses will be required to establish causality. Our data do not establish mechanistic identity across diseases; they align morphologically with patterns described in other contexts.

### A unifying biological principle: a hypothesis of pathological regression

We hypothesize that this conserved pathophenotype reflects a partial regression of the NVU toward a developmentally less mature, pro-angiogenic state. This hypothesis is consistent with prior reports showing pathological shifts that mimic developmental processes. For example, the switch from low to high endothelial transcytosis is a reversion to a more permeable phenotype^23,24^. The loss of astrocytic AQP4 polarity, documented in various CNS pathologies, represents a de-specialization of their mature function^25^. In gliomas, a switch from quiescent to pro-invasive laminin isoforms in the basement membrane is a direct reversion to a matrix composition characteristic of development^26^. While our TEM data cannot resolve molecular programs, they motivate quantitative, multi-omic tests of this regression framework.

### From microscopic structure to macroscopic function

The conserved remodeling of the NVU has profound clinical consequences, including the heterogeneity of the resulting BTB^27^. Advanced imaging techniques like DCE-MRI provide a macroscopic window into these microscopic changes by quantifying vascular permeability (*K*^*trans*^)^28^. Our framework suggests that the functional heterogeneity seen in clinical imaging is not random noise but the macroscopic manifestation of this underlying regression process occurring asynchronously in space. Regions with elevated DCE-MRI *K*^*trans*^ may correspond to areas with more advanced NVU remodeling, whereas non-enhancing edges may reflect a relatively intact barrier where tumor cells have co-opted existing vessels^29,30^; this structure–function linkage remains to be tested with spatially co-registered datasets.

### Therapeutic implications of model selection

Although our ultrastructural analysis revealed a conserved pathological phenotype in established, macroscopic lesions regardless of the seeding route, the functional response to chemotherapy is expected to diverge due to the distinct distributional landscapes of the two models. The IC model generates a unifocal, bulk tumor mass that typically mimics the high-permeability state of advanced disease. In contrast, the m-ICA model recapitulates a heterogeneous spectrum of metastatic progression, characterized by the coexistence of macroscopic lesions and numerous micrometastases. Since blood-tumor barrier permeability is often size-dependent—with micrometastases retaining a relatively intact BBB compared to bulkier tumors^29^—we anticipate that the m-ICA model would yield lower overall response rates to systemic therapies that have poor BBB penetrance. Consequently, while the IC model may be sufficient for evaluating efficacy against established bulk disease, the m-ICA platform offers a more rigorous test for therapeutic agents targeting the entire natural history of brain metastasis, including the critical prevention of micrometastatic outgrowth.

### Limitations and future directions

First, regarding model selection, we utilized 4T1 and B16 cell lines because they are syngeneic to immunocompetent mice, preserving the critical role of the immune system in NVU remodeling. However, this study was restricted to one cell line per tumor type (TNBC and melanoma) and did not include HER2 + breast cancer models or patient-derived xenografts (PDX). Consequently, the generalizability of our findings to other molecular subtypes or to human biology remains to be established. Second, the standard ICA model was excluded from ultrastructural analysis. As demonstrated in Fig. 1, the severe extracranial burden in the standard ICA model often necessitated early humane euthanasia, preventing the reliable collection of established brain lesions comparable to the m-ICA and IC groups. Third, ultrastructural analyses were performed on representative specimens (specifically the B16 model, to leverage pigment-assisted targeting) without stereological sampling, immunogold labeling, or formal blinded scoring. Fourth, OS analyses were not powered for equivalence or non-inferiority; non-significant differences should not be interpreted as evidence of equality.

Future work will test this framework by integrating spatial transcriptomics with co-registered permeability imaging (e.g., DCE-MRI K^trans^) and stereology-based EM, enabling quantitative structure–function modeling. Prospective registration and power analyses will pre-specify primary endpoints and minimally detectable effects.

In conclusion, our work provides a methodologically refined platform for brain metastasis research and offers direct ultrastructural evidence to support a conserved BTB pathology in established lesions, regardless of seeding route. We further place this observation within a testable conceptual framework of pathological regression. This provides a new perspective on the pathophysiology of brain metastasis and charts a clear course for future quantitative and mechanistic investigation.

## Methods

### Cell lines and culture

The murine breast cancer cell line 4T1 (RRID: CVCL_0125) and melanoma cell line B16-F10 (RRID: CVCL_0159), both stably expressing firefly luciferase, were obtained from the American Type Culture Collection (ATCC, Manassas, VA, USA). Cells were cultured in RPMI-1640 medium (Gibco) supplemented with 10% fetal bovine serum (FBS; Gibco) and 1% penicillin–streptomycin (Gibco) at 37 °C with 5% CO₂. Cell identity was confirmed by Short Tandem Repeat (STR) profiling, and cultures were routinely tested and confirmed to be mycoplasma-free prior to experiments.

### Animal studies and ethical compliance

All animal experiments were performed in strict accordance with a protocol approved by the Animal Research Ethics Committee of West China Hospital, Sichuan University. Female BALB/c (for 4T1 cells, weighing approximately 20–22 g) and C57BL/6 (for B16-F10 cells, weighing approximately 18–20 g) mice, 8 weeks of age, were acquired from Beijing HFK Bioscience Co., Ltd. (Beijing, China) and housed under specific pathogen-free conditions with ad libitum access to food and water. Mice were randomly allocated to experimental groups. Investigators acquiring in vivo imaging were blinded to group allocation. Humane endpoints were defined as > 20% body weight loss or the onset of severe neurological deficits, at which point animals were euthanized by CO₂ inhalation. For animals reaching the pre-defined experimental endpoint for tissue analysis, they were transcardially perfused under deep pentobarbital anesthesia as described in the 'Histology and Ultrastructural Analysis’ section.

### Establishment of brain metastasis models

All surgical procedures were performed under anesthesia (pentobarbital sodium, 50 mg/kg, i.p.). Ophthalmic ointment was applied to prevent corneal drying, and body temperature was maintained with a heating pad.

Conventional Intracarotid Artery (ICA) Injection: As a baseline for methodological comparison, the right common carotid artery (CCA) was surgically exposed, and a suspension of 1 × 10^5^ tumor cells in 100 µL of serum-free medium was slowly injected.

Modified Intracarotid Artery (m-ICA) Injection: To minimize extracranial seeding, after surgical exposure of the carotid bifurcation, the external carotid artery (ECA) was permanently ligated with 6–0 silk. Subsequently, 1 × 10^5^ tumor cells in 100 µL of serum-free medium were injected into the CCA over approximately 60 s.

Stereotactic Intracranial (IC) Inoculation: Mice were placed in a stereotactic frame. A burr hole was drilled at AP 0 mm, ML + 2.0 mm relative to bregma. A 33-G needle delivered 5 × 10^4^ tumor cells in 2 µL of serum-free medium at a depth of DV 3.0 mm at a rate of 0.2 µL/min. The needle was left in place for 2 min before slow withdrawal to minimize reflux.

### In vivo imaging

Bioluminescence Imaging (BLI): Based on kinetic studies establishing an optimal acquisition window (Supplementary Fig. S1), D-luciferin (150 mg/kg, i.p.) was administered 10 min before imaging. Imaging was performed on an IVIS Spectrum system (PerkinElmer) under 2% isoflurane with standardized settings. A standardized circular region of interest (ROI) encompassing the cranium was used to quantify photon flux (photons/s) using Living Image software (PerkinElmer).

Magnetic Resonance Imaging (MRI): Non-contrast T2-weighted MRI of the head was acquired on a 7.0-T small-animal MRI scanner (Bruker) to visualize extracranial tumor masses (TR/TE 2500/33 ms; slice thickness 0.5 mm; matrix 256 × 256; FOV 20 × 20 mm).

### Histology and ultrastructural analysis

For histology, mice were transcardially perfused with PBS followed by 4% paraformaldehyde (PFA) at the experimental endpoint. Brains were harvested, post-fixed, embedded in paraffin, and coronal sections were stained with hematoxylin and eosin (H&E).

For transmission electron microscopy (TEM), a targeted, qualitative analysis was performed on B16 melanoma brain metastases. The B16 model was selected for this ultrastructural interrogation because the intrinsic melanin pigment of the tumor cells served as a critical visual guide for precise localization of the tumor-brain interface within resin blocks, a step that is technically challenging with non-pigmented 4T1 cells. At the endpoint, representative mice (one from the m-ICA model and one from the IC model) were transcardially perfused with 2.5% glutaraldehyde. This exploratory analysis was performed on n = 1 per model to illustrate recurring features at the tumor–brain interface; no stereological sampling or immunogold labeling was performed. Tissue blocks from pigmented tumor regions were fixed, post-fixed in 1% osmium tetroxide, embedded in Epon 812 resin, and sectioned into ultrathin slices (≈70–90 nm). Sections were stained with uranyl acetate and lead citrate and imaged on a Hitachi H-7650 TEM. Multiple microvascular fields were examined per specimen under qualitative, consensus-based assessment. Accordingly, ultrastructural findings are hypothesis-generating and not intended for unbiased quantification or molecular localization.

### Statistical analysis

Statistical analyses were performed using GraphPad Prism (Version X, GraphPad Software, San Diego, CA, USA). BLI signals were log-transformed. Slopes representing growth rates were compared using an overall F-test on the group × time interaction; individual fits were performed per animal. Kaplan–Meier survival curves were compared by the log-rank test. A two-sided *P* < 0.05 was considered statistically significant. No equivalence or non-inferiority testing was performed. Survival analyses were not adjusted for multiple comparisons and should be interpreted as failure to reject the null under current cohort sizes and humane endpoints.

## Supplementary Information

Below is the link to the electronic supplementary material.

Supplementary Material 1

Supplementary Material 2

## Data Availability

All data generated or analyzed during this study are included in this published article and its Supplementary Information files. The source data for tumor burden and body weights are provided in Supplementary Data 1.
