# Supplementary material for: Route-dependent dissemination with conserved blood–tumor barrier ultrastructure in intracranial metastasis models
Source: Sci Rep. 2026 Mar 14;16:13508. doi: 10.1038/s41598-026-37760-z (PMC13111581; doi:10.1038/s41598-026-37760-z)
Supplement: Supplementary file 2 — Supplementary Material 2 [file 41598_2026_37760_MOESM2_ESM.pdf]

Article Title: Route-dependent Dissemination with Conserved Blood–Tumor Barrier Ultrastructure in Intracranial Metastasis Models
Journal: Brain Structure and Function
Authors: Jian Zhao[1], Yuehua Zhang[2], Zhigong Wei[3], Kai Li[1], Dan Li[4]*, Yongsheng Wang[1]*
Corresponding Author: Dan Li, MD Affiliation: Institute of Respiratory Health, Frontiers Science Center for Disease-related Molecular Network, and Precision Medicine Research Center, Precision Medicine Key Laboratory of Sichuan Province, West China Hospital, Sichuan University, Chengdu, Sichuan 610041, P.R. China. Email: lidan@wchscu.cn
Yongsheng Wang, MD Affiliation: Thoracic Oncology Ward, Cancer Center, and State Key Laboratory of Biotherapy, West China Hospital, Sichuan University, Chengdu, Sichuan, 610041, P.R. China. Email: wangys@scu.edu.cn

Supplementary Figure S1: Optimization and validation of the bioluminescence imaging time-window.

To determine the optimal time for signal acquisition, the kinetics of bioluminescence were monitored following intraperitoneal injection of D-luciferin in mice bearing intracranial tumors of different sizes, representing low and high tumor burdens. (a) Representative time-series of in vivo BLI in mice with 4T1 breast cancer tumors at day 5 (low burden, top row) and day 20 (high burden, bottom row) post-inoculation. Images were acquired every 2-5 minutes for 40 minutes. (b) Quantification of the bioluminescent signal from the region of interest (ROI) over time for the 4T1 model, showing that peak signal intensity is reached between 10 and 15 minutes post-injection for both low (blue line) and high (red line) tumor burdens. (c) Representative time-series of in vivo BLI in mice with B16 melanoma tumors at day 5 (low burden, top row) and day 15 (high burden, bottom row) post-inoculation. (d) Quantification of the bioluminescent signal over time for the B16 model, similarly demonstrating a consistent signal peak between 10 and 15 minutes. These data confirm that the 10–15 minute post-injection time point is optimal and reproducible for quantifying tumor burden, irrespective of the cell line or tumor size, and validate its use for all longitudinal imaging studies presented.

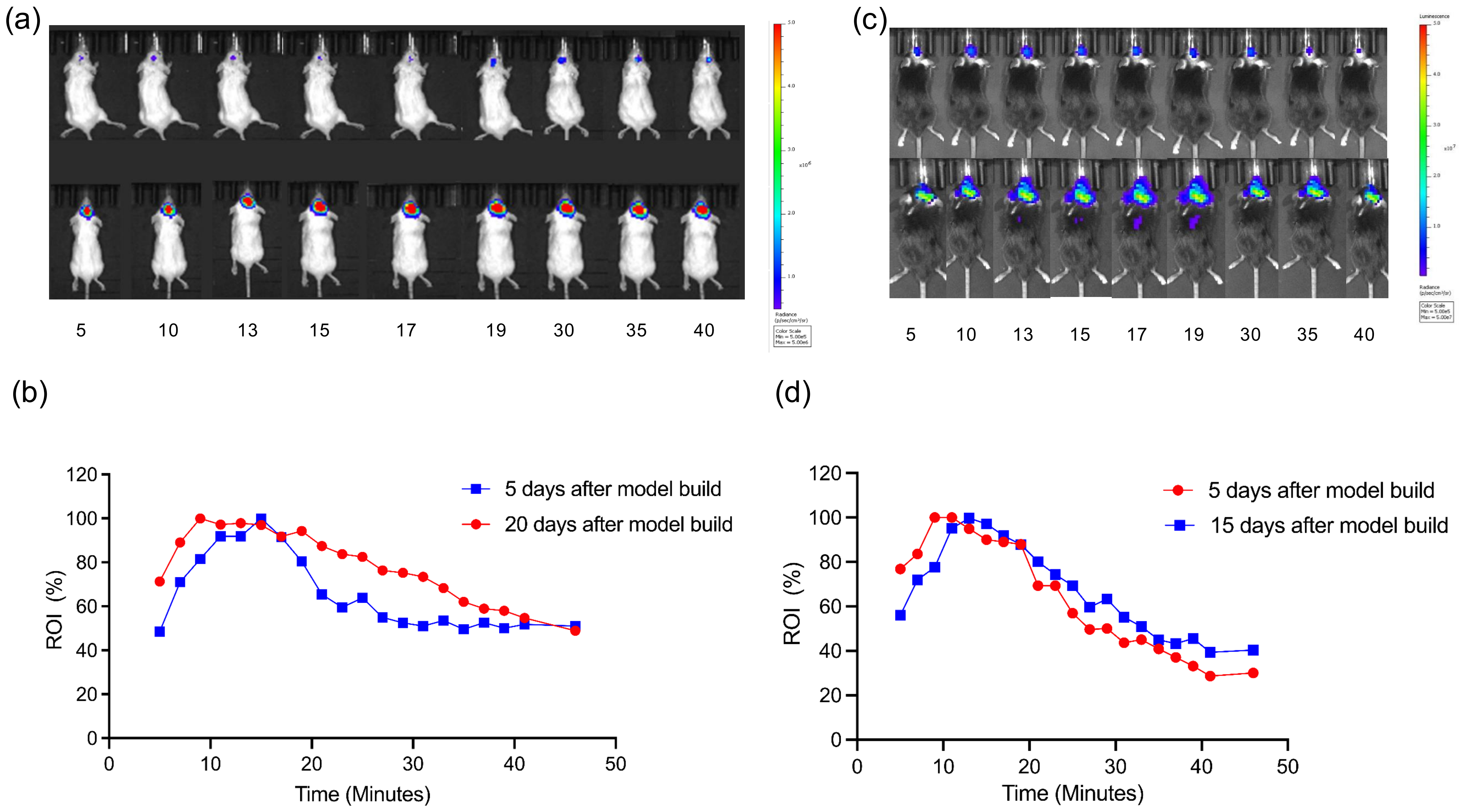

(a)
5
10
13
15
17
19
30
35
40
(c)
5
10
13
15
17
19
30
35
40
(b)
5 days after model build
20 days after model build
ROI (%)
Time (Minutes)
(d)
5 days after model build
15 days after model build
ROI (%)
Time (Minutes)
